# The myth of brain damage: no change of neurofilament light chain during transient cognitive side-effects of ECT

**DOI:** 10.1007/s00406-023-01686-8

**Published:** 2023-09-01

**Authors:** Matthias Besse, Michael Belz, Claudia Bartels, Bettina Herzig, Jens Wiltfang, David Zilles-Wegner

**Affiliations:** 1 Department of Psychiatry and Psychotherapy, University Medical Center Göttingen, Von-Siebold-Strasse 5, 37075 Göttingen, Germany; 2 German Center for Neurodegenerative Diseases (DZNE), Goettingen, Germany; 3 Neurosciences and Signaling Group, Institute of Biomedicine (iBiMED), Department of Medical Sciences, University of Aveiro, Aveiro, Portugal

**Keywords:** Electroconvulsive therapy (ECT), Neurofilament light chain (NfL), Brain damage, Biomarkers, Cognition, Neuropsychology

## Abstract

Electroconvulsive therapy (ECT) is an effective, safe, and mostly well-tolerated treatment for patients with severe or difficult to treat depression or psychotic disorders. However, a relevant number of patients experience subjective and/or objective cognitive side-effects. The mechanism of these transient deficits is not yet clear. Thus, our study prospectively investigated neurofilament light chain (NfL) concentrations as a highly sensitive biomarker for neuroaxonal damage along with cognitive performance during a course of ECT. Serum NfL concentrations from 15 patients with major depressive disorder receiving ECT were analyzed (1) 24 h before the first ECT, (2) 24 h and (3) 7 days after the last ECT (45 measurements in total). Neuropsychological testing including memory, executive functions and attention was performed at each time-point. NfL concentrations did not change between the three time-points, while a temporary cognitive impairment was found. Even in the subset of patients with the strongest impairment, NfL concentrations remained unchanged. Neuropsychological testing revealed the common pattern of transient cognitive side-effects with reduced performance 24 h post-ECT (global cognition score: *p* < 0.001; memory: *p* = 0.043; executive functions: *p* = 0.002) and return to baseline after 7 days (all *p* < 0.001). Our study adds to the evidence that neither ECT per se nor the transient cognitive side-effects seem to be associated with an increase of NfL as a marker of neuroaxonal damage. In contrast, we discuss cognitive side effects to be potentially interpreted as a byproduct of ECT’s neuroplastic effects.

**Supplementary Information:**

The online version contains supplementary material available at 10.1007/s00406-023-01686-8.

## Introduction

Electroconvulsive therapy (ECT) is a highly effective treatment for severe affective and psychotic disorders [1]. It is well tolerated by the vast majority of patients, and can be considered a safe and reliable procedure.

Common side-effects of ECT include nausea, headache and cognitive impairment. The latter is mainly characterized by impairments of episodic memory as well as executive functioning. Usually, these side-effects are considered to be transient and resolve completely within a maximum of two weeks after the last ECT treatment [2]. Furthermore, even an ongoing treatment with maintenance-ECT (mECT) does not affect cognitive performance [3].

Despite its safety profile and clear evidence concerning reversibility of side-effects (see above), some patients are specifically worried about cognitive impairment and possible “brain damage”. As a consequence, some patients might reject a highly promising treatment for their severe psychiatric disorder [4–6]. Few authors still claim that ECT would cause structural brain damage and/or neuronal loss, leading to permanent cognitive impairment [7], thus supporting unjustified ECT-related anxiety. Up to now, different study types including neuroimaging studies with structural magnetic resonance imaging (MRI) and positron emission tomography (PET) [8, 9], human post-mortem autopsy [10], or the analysis of molecular markers in blood or cerebrospinal fluid (CSF) of ECT-treated patients [11, 12] did not find any evidence supporting structural brain damage. On the contrary, a rising amount of studies report a grey matter volume increase in different areas of the brain (hippocampus, amygdala striatum) associated with ECT [8, 13, 14].

Despite this evidence, one could still argue that these methods are not sensitive enough to detect more subtle disruptive effects [13] and to rule out that cognitive side-effects, although transient, might, however, be associated with subtle increases of biomarkers of neural damage like neurofilament light chain (NfL). Neurofilaments, consisting of light, medium and heavy chains, are structural proteins of the axonal cytoskeleton in the central nervous system (CNS). Even the slightest, non-imaging detectable damage in the CNS leads to a release of NfL into the patient’s cerebrospinal fluid and blood, making NfL one of the most sensitive biomarkers of neuronal damage [15]. By using the ultrasensitive SIMOA technology [16], NfL has been established as a sensitive biomarker in diagnosis and monitoring of different neurological diseases, like multiple sclerosis [17], acute ischemic stroke [18], traumatic injury of the brain [19], or dementia [20]. Thus, this biomarker is well suited to detect even subtle negative impacts, potentially affecting neuronal tissues.

In a previous study, our group demonstrated that NfL concentrations in the peripheral blood remained stable over a course of ECT [21]. However, this study used the Mini-Mental State Examination (MMSE) to monitor cognitive performance only, which is not very sensitive and thus did not detect any cognitive deficits during the course of ECT [22].

The aim of the present study was to combine a most sensitive and longitudinal analysis of NfL as primary outcome with a more profound and sensitive neuropsychological testing (secondary outcome). Our hypothesis was that ECT-associated cognitive side-effects can occur despite stable intrapersonal variations in NfL concentrations.

## Methods

### Subjects

A total of *N* = 15 patients at the Department of Psychiatry and Psychotherapy, University Medical Center Göttingen, were included in this study between 09/2020 and 01/2022. They were between the ages of 25 and 77 (*M* = 56.93, SD = 14.93) and female by majority (73.3%, *n* = 11). The following inclusion criteria were applied: (1) clinical indication for ECT treatment, (2) minimum age of 18 years, and (3) at least moderate to severe unipolar depressive episode (ICD-10: F32.1–F32.3 and F33.1–F33.3). Patients were excluded, if they suffered from (1) dementia (ICD-10: F00–F03), (2) organic affective disorders (ICD-10: F06.3) or (3) current substance dependence (except for tobacco; ICD-10: F10–F16, F18–F19). There were no restrictions regarding concomitant medication (see Table 1 for medication details), but drugs had to be kept unchanged throughout the study. The study was approved by the ethics committee of the University Medical Center Göttingen (ethical vote 21/6/14). Written informed consent was obtained from all participants prior to the study.

**Table 1:** Medication

	Antidepressant	Antipsychotic	Mood stabilizer
SSNRI	3	–	–
SSRI	2	–	–
Tricyclic	1	–	–
Other	4	–	–
Combination	5	–	–
None	0	–	–
Atypical	–	6	–
Combination	–	4	–
None	–	5	–
Lithium	–	–	3
Lamotrigine	–	–	0
None	–	–	12

Medication for *N* = 15 patients

### Study design

Change in NfL concentrations has been predefined as primary outcome, comprising 3 measurements per patient, 45 measurements in total: blood samples were collected within 24 h prior to the first treatment (pre-ECT: *T*_1_), within 24 h after the last ECT session (post-ECT: *T*_2_), and 1 week after the last ECT session (follow-up; *T*_3_), due to the known dynamics of NfL with at least a few days between exposure and the peak of NfL concentrations in the blood [23]. Besides NfL concentration, cognitive parameters (see below) were assessed in the same visits as secondary outcomes, along with depression severity as measured by MADRS and BDI-II.

### ECT treatment

A Thymatron IV device (Somatics, LLC., Lake Bluff, IL, USA) was used, applying the brief pulse technique and the double-dose program (maximum dose of 1008mC, 200%). Age-based dosing was used in the initial session, dosage was then adjusted depending on clinical response as well as seizure quality (mean dosage of *M* = 73.60%, SD = 29.9%). Patients received *M* = 10.80 (SD = 2.96) ECT sessions. In this sample, electrode placement with right unilateral (*n* = 1) and left anterior right temporal (LART) position (*n* = 11) was chosen. In *n* = 3 patients, electrode placement was adjusted according to response and tolerability. In all cases, a combination of propofol and esketamine was used for anesthesia and succinylcholine was used as muscle relaxant.

### Blood sampling and NfL measurements

Blood was collected at three different time-points (see above). Serum was stored for 45 to 60 min at room temperature to await coagulation and processed afterwards according to the local standard operating procedures. Briefly, the serum was centrifuged at 2000 × *g* for 10 min at room temperature, aliquoted as 500 µl samples and stored at − 80 °C.

NfL concentration (pg/ml) was measured according to the manufacturers protocol (Simoa, #102258).

### Cognitive parameters

Besides MMSE, neuropsychological tests were selected to allow a more sensitive detection of changes in cognitive performance during the course of ECT. They were composed to cover cognitive domains most susceptible to transient ECT side-effects (memory, executive functions, attention) [2] and comprised to following well-established cognitive tests: (1) memory: German version of the Rey Auditory Verbal Learning Test (RAVLT, sum of learning trials 1–5, delayed recall); (2) executive functions: categorical (animals) and phonemic (s-words) fluency of the Regensburg Word Fluency Test (RWT), Trail Making B (TMT B), Digit Span backwards of the Wechsler Adult Intelligence Scale–fourth edition (WAIS-IV), and (3) attention: Trail Making A (TMT A), WAIS-IV Digit Span forward. To avoid overrepresentation of single test scores, cognitive variables entered analyses as cognitive domain scores based on means of standardized T scores (memory score, executive score, attention score). Besides the three cognitive domain scores, the (4) global cognition score was calculated as mean of standardized T scores from all cognitive tests. Raw scores for cognitive variables and for each testing time-point are presented in Supplementary Table S1. All neuropsychological examinations were performed by experienced and trained psychiatrists or neuropsychologists in a standardized manner.

### Statistical analyses

To calculate the required sample size for this study a priori regarding our primary outcome (concentration of NfL), G*Power (F. Faul, University of Kiel, Germany) was used. Three repeated measurements per patient (*T*_1_: pre-ECT, *T*_2_: post-ECT, *T*_3_: follow-up) were anticipated for general linear modelling (GLM), given *α* = 0.05 and 1 − *β* = 0.95. A medium effect size (*f* = 0.25) was chosen due to limited availability of empirical data on NfL concentrations under ECT so far [24]. As shown by multiple authors, NfL levels are assumed to be highly correlated on intra-individual level (e.g., [21, 23, 25]). To calculate the required sample size, we, therefore, set the correlation among repeated measures of NfL concentration as *r* = 0.85, which was later empirically confirmed in our sample (*r* = 0.98, *p* < 0.001). Overall, the calculations with G*Power led to a minimal sample size of *N* = 14 (42 repeated measurements) which was exceeded by one patient (*N* = 15, 45 repeated measurements).

IBM SPSS Statistics 29 (IBM Corp. Armonk, NY) was used to analyze data. For numeric variables, we created means (*M*) and standard deviations (SD). For within-sample analyses, we created multiple general linear models (GLM) for repeated measures, adding dependent variables as three-staged within-individual factors, both for our primary outcome (concentration of NfL) and secondary outcomes (four cognitive domain scores, MMSE, MADRS, BDI-II). For multiple comparisons, *p* values were corrected using the Bonferroni method both within the GLM for NfL, and within all GLMs for our secondary outcomes (initial significance was set at *p* < 0.05). Besides eta-square (η^2^) as effect size for overall variation of repeated measurements within each GLM, we also calculated Cohen’s d (*d*) for all separate pairwise comparisons between single measurements to further evaluate the strength of those pairwise differences (*T*_1_ vs. *T*_2_; *T*_1_ vs. *T*_3_; *T*_2_ vs. *T*_3_). All levels of significance are reported two-tailed. Missing values did not occur for any of the dependent variables, except for the MADRS which was available at all measurements for 11 out of 15 patients (33 measurements in total).

## Results

### NfL concentration

**Fig. 1:**
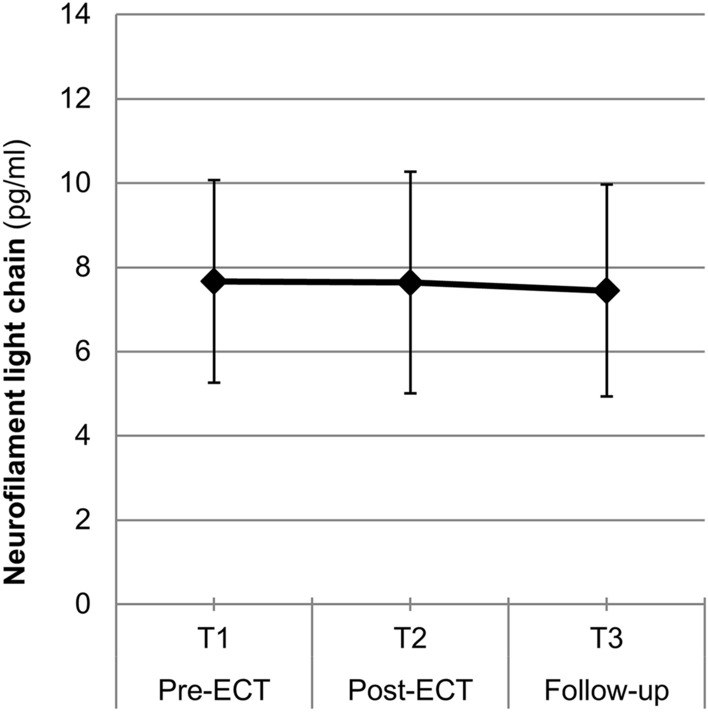
*p < 0.05, **p < 0.01, ***p < 0.001. Mean values with 95%-CIs; pre-ECT (*T*_1_), post-ECT (*T*_2_) and follow-up measurement (*T*_3_); neurofilament light chain concentration (pg/ml); *N* = 15

We found minimal numerical changes in NfL concentrations (pg/ml) between pre-ECT (*T*_1_: *M* = 7.67, SD = 4.76), post-ECT (*T*_2_: *M* = 7.64, SD = 5.20), and follow-up measurement (*T*_3_: *M* = 7.45, SD = 4.97; see Fig. 1). The GLM did not reveal any significant overall variation (*F*(2, 28) = 0.32, *p* = 0.713, partial *η*^2^ = 0.02, *ns*), nor significant differences between measurements (all Bonferroni corrected pairwise comparisons: *p* = 1.00, ns., *d* from 0.02 to 0.22). To avoid a possible type II error caused by traditional two-sided testing (e.g., [26, 27]), we calculated three separate t-tests for repeated measures to compare each pair of measurements excluding Bonferroni correction without any significant differences between pre-, post-, and follow-up-measurements (*t*(14) = 0.09 to 0.87, *p* = 0.402 to 0.933, ns.).

### Cognitive parameters

**Fig. 2:**
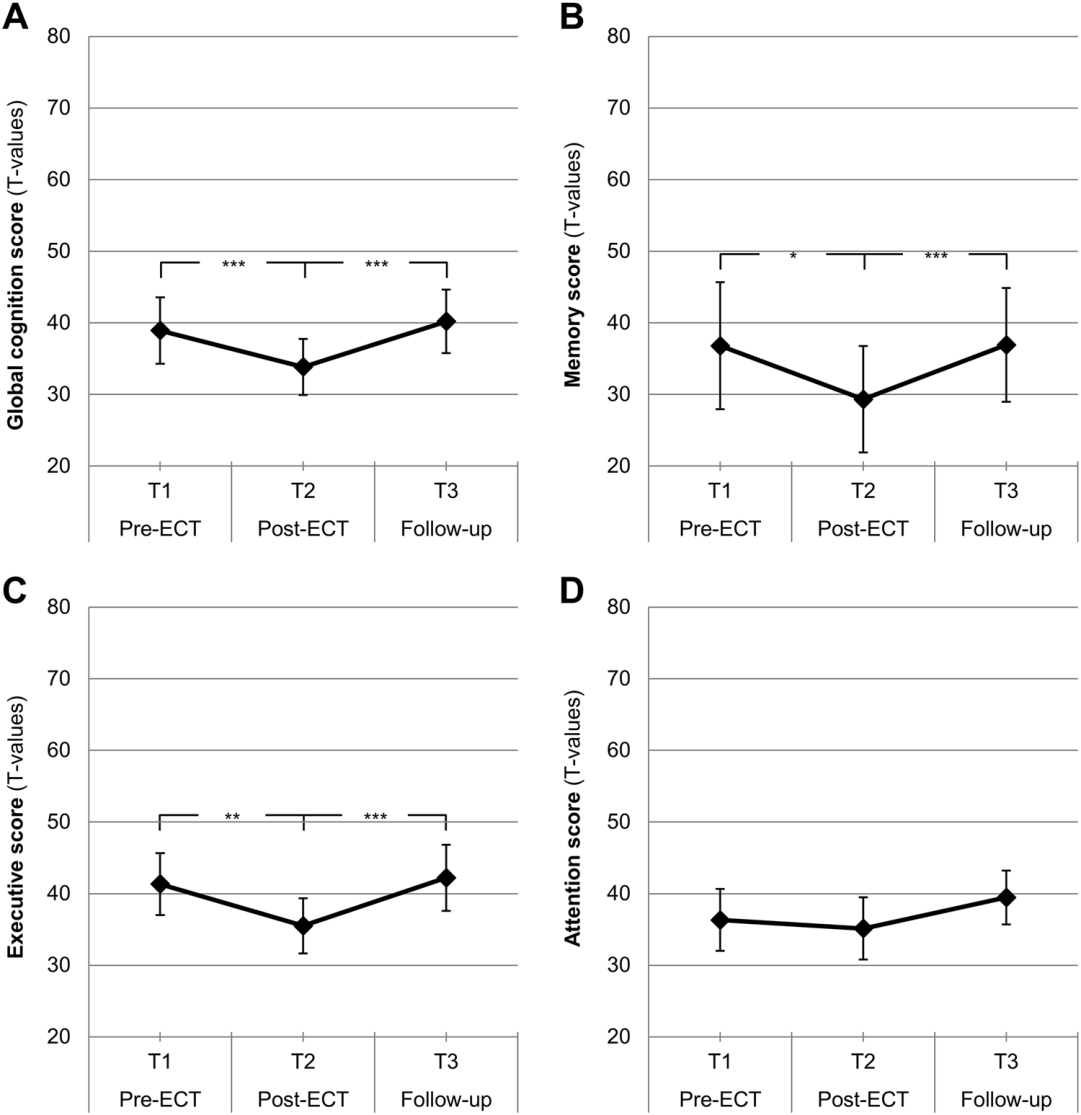
*p < 0.05, **p < 0.01, ***p < 0.001. Mean values with 95%-CIs; pre-ECT (*T*_1_), post-ECT (*T*_2_) and follow-up measurement (*T*_3_), arithmetic mean of *T* values for cognitive parameters: **A** global cognition score; **B** memory score; **C** executive score; **D** attention score. *N* = 15

Please see Fig. 2 and Table 2 for a summarization of the results for the four compound scores. A significant decrease in cognitive scores from pre- to post-measurement (*T*_1_ to *T*_2_) was found for three out of four compound scores: (1) global cognition score (*p* < 0.001), (2) memory score (*p* = 0.043), and (3) executive score (*p* = 0.002). These three cognitive parameters subsequently showed a normalization at follow-up (*T*_2_ to *T*_3_, all *p* < 0.001), with all values numerically exceeding the baseline scores (see Fig. 2 and Table 2 for details). The (4) attention score also declined from pre- to post-measurement (*T*_1_ to *T*_2_), and improved from post- to follow-up measurement (*T*_2_ to *T*_3_), but changes did not reach significance (*p* from 0.070 to 0.999). Overall, short-term cognitive side-effects could be detected which were completely reversed 1 week after the last ECT.

**Table 2:** Cognitive parameters

Cognition scores	Pre-ECT (*T*_1_)	Post-ECT (*T*_2_)	Follow-up (*T*_3_)	GLM (*F*_(2, 28)_, *p*, *η*^2^)	*T*_1_ vs. *T*_2_	*T*_1_ vs. *T*_3_	*T*_2_ vs. *T*_3_
Global cognition	*M* = 38.93 ± 9.16	*M* = 33.85 ± 7.74	*M* = 40.20 ± 8.75	*F* = 18.52, *p* < .001, *η*^2^ = 0.57	*p* < .001, *d* = 1.29	*p* = .999, *d* = 0.24	*p* < .001, *d* = 1.85
Memory	*M* = 36.80 ± 17.53	*M* = 29.33 ± 14.64	*M* = 36.93 ± 15.68	*F* = 8.08, *p* = .002, *η*^2^ = 0.37	*p* = .042, *d* = 0.73	*p* = .999, *d* = 0.02	*p* < .001, *d* = 1.32
Executive	*M* = 41.33 ± 8.56	*M* = 35.52 ± 7.61	*M* = 42.21 ± 9.12	*F* = 10.28, *p* < .001, *η*^2^ = 0.42	*p* = .002, *d* = 1.15	*p* = .999, *d* = 0.12	*p* < .001, *d* = 1.19
Attention	*M* = 36.33 ± 8.52	*M* = 35.13 ± 8.60	*M* = 39.47 ± 7.47	*F* = 2.29, *p* = .120, *η*^2^ = 0.14	*p* = .999, *d* = 0.13	*p* = .506, *d* = 0.38	*p* = .070, *d* = 0.66

*T* values with respective *M* = mean ± standard deviation; *GLM*: *F*-/*p*-/ η2-values for repeated measures effect; Bonferroni corrected pairwise comparisons (e.g., *T*_*1*_ vs. *T*_*2*_) are reported with *p* values and Cohen’s *d*

Important to note from a clinical perspective, pre-ECT cognitive performance was below normal (*T* scores < 40) for global cognition, memory and attention. Subsequently, cognitive performance further decreased post-ECT and improved at follow-up to even reach the normal range (*T* scores ≥ 40) in global cognition and executive functions (see Table 2).

**Fig. 3:**
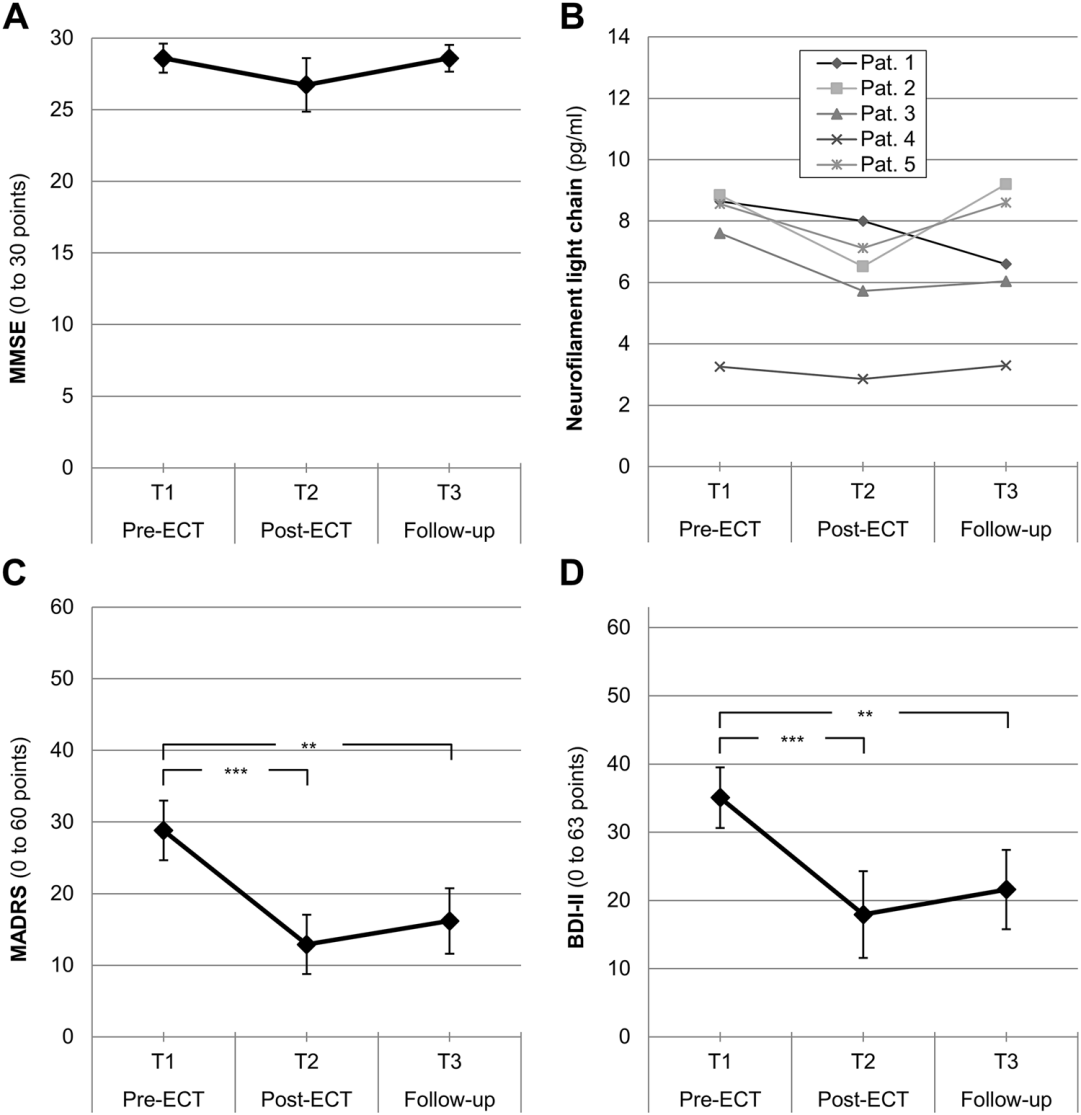
*p < 0.05, **p < 0.01, ***p < 0.001. Mean values with 95%-CIs; pre-ECT (*T*_1_), post-ECT (*T*_2_) and follow-up measurement (*T*_3_); **A** MMSE (0 to 30 points, *N* = 15); **B** Neurofilament light chain concentration for *n* = 5 patients with strongest cognitive impairment (global cognition score); **C** MADRS (0–60 points, *N* = 15); **D** BDI-II (0–63 points, *N* = 15)

The MMSE score generally varied over three measurements (*F*(2, 28) = 5.47, *p* = 0.010, partial *η*^2^ = 0.28, see Fig. 3A), but pairwise comparisons failed to reach significance (*p* from 0.090 to 0.999, *d* from 0.01 to 0.76). Numerically, patients showed an impairment from pre-ECT (*T*_1_: *M* = 28.60, SD = 1.99) to post-ECT (*T*_2_: *M* = 26.73, SD = 3.71), and a normalization at follow-up measurement (*T*_3_: *M* = 28.60, SD = 1.84). However, MMSE mean values for all assessment time-points essentially fell within normal limits (i.e. MMSE scores ≥ 27).

### Additional analysis: NfL concentrations and cognitive side-effects

To further investigate if the transient cognitive side-effects reported above were related to changes in NfL concentrations within our sample, we created a subsample of *n* = 5 patients who showed the strongest decrease in the global cognition score from pre-ECT to post-ECT measurement (*T* value delta from – 8.62 to – 10.75, Δ*M* = − 9.75, SD = 0.86). Even in this subsample, there was no increase of NfL concentrations. From pre-ECT to post-ECT measurement, all NfL concentrations numerically decreased (Δ*M* = − 1.34 pg/ml). From post-ECT to follow-up measurement, the majority of NfL concentrations increased (Δ*M* = 0.70 pg/ml), but NfL concentrations at follow-up (*M* = 6.75 pg/ml) still were relatively lower when compared to pre-measurement (*M* = 7.38 pg/ml; Fig. 3B).

### Effectiveness of ECT

Depressive symptoms significantly varied from pre-ECT over post-ECT to follow-up measurement, both for MADRS (*T*_1_: *M* = 28.82, SD = 7.05; *T*_2_: *M* = 12.91, SD = 7.02; *T*_3_: *M* = 16.18, SD = 7.76; GLM: *F*(2, 20) = 21.22, *p* < 0.001, partial *η*^2^ = 0.68; see Fig. 3C) and for BDI-II (*T*_1_: *M* = 35.07, SD = 8.82; *T*_2_: *M* = 17.93, SD = 12.60; *T*_3_: *M* = 21.60, SD = 11.46; GLM: *F*(2, 28) = 14.29, *p* < 0.001, partial *η*^2^ = 0.50; see Fig. 3D).

Both the reductions from *T*_1_ to *T*_2_ (MADRS: Δ*M* = − 15.91 points, *p* < 0.001, *d* = 1.85; BDI-II: Δ*M* = − 17.13 points, *p* < 0.001, *d* = 1.17), and *T*_1_ to *T*_3_ (MADRS: Δ*M* = − 12.64 points, *p* = 0.005, *d* = 1.27; BDI-II: Δ*M* = − 13.47 points, *p* = 0.005, *d* = 1.01) were significant.

## Discussion

This is the first study to investigate both the course of NfL during a series of ECT and the possible association of NfL dynamics with the degree of cognitive side-effects. For both NfL and cognition, we used highly sensitive methods with the capability to detect even small changes in the respective area. While we found the frequently replicated trajectory of cognitive side-effects (a short-term decrease with a rapid return or even improvement in comparison to baseline levels [2]), there was neither a simultaneous change, nor a significant change at all in NfL concentrations between measurements. In sum, neither ECT per se nor the transient cognitive side-effects were associated with an increase of NfL as a sensitive marker of neuroaxonal damage [15]. These results in part replicate and extend previous studies [11, 12, 21, 28–32] on the issue of biomarkers for neuronal damage in the context of ECT.

Extending our previous study, we implemented a cognitive test battery inspired by the detailed meta-analysis of Semkovska et al. [2]. Using this comprehensive neuropsychological test battery, we detected a significant short-term decrease in cognitive performance from pre-to post-ECT in three out of four cognitive parameters (memory, executive functions, global cognition). In the follow-up measurement, 1 week after the last ECT, all cognitive parameters revealed a normalization towards the respective baseline level. These results underscore the sensitivity of the cognitive test battery to track cognitive changes. It is also important to note that our study sample showed cognitive impairments (*T* scores < 40) in the pre-ECT measurement (global cognition, memory and attention), as might be expected for patients with severe depressive symptoms [33, 34], while pre-ECT MMSE scores did not indicate any significant impairment. This finding further underlines a higher sensitivity of our cognitive test battery to also detect cognitive impairment compared to the MMSE [21]. In terms of clinical relevance of these changes, it is also worthy of note that cognitively impaired patients at pre-ECT finally returned to performance levels in the (low) normal range at follow-up (*T* scores > 40), as summarized by the global cognition score. This finding once more suggests the absence of long-term cognitive side-effects in the treatment course of ECT, or vice versa implies that ECT-induced remission of depressive symptoms is paralleled by cognitive improvement.

Although NfL and cognitive impairment were not related in the total sample, we further selected a subsample of five patients with the strongest cognitive side-effects. Within these, NfL also remained stable over the course of ECT. Thus, even marked cognitive side-effects in some patients do not seem to be associated with increased levels of NfL.

Yet an ECT-induced temporary cognitive impairment from pre- to post-measurement reflects the existing literature in this field: it has also been reported by recently published studies, including a variety of neuropsychological tests like the Screen for Cognitive Impairment in Psychiatry (SCIP) and Trail Making Test-Part B (TMT-B) [4] or the Montreal Cognitive Assessment (MoCA) [35]. There, an impairment was usually found within a few days up to 1 week post-ECT, while no long-term cognitive side-effects of ECT could be observed (see also [36]). These results raise the question of the mechanism of ECT-induced temporary cognitive impairment. As neuronal damage does not seem to be the cause of temporary impairment, other aspects must play a pivotal role. While our study does not provide any conclusive answers regarding this specific question, a growing body of literature reports significant grey matter volume increases following ECT, especially a pronounced enlargement of the hippocampus, [8, 37–41]. Several recent studies found a correlation between ECT-induced hippocampal volume increase and cognitive impairment [42, 43]. The cognitive side-effects may thus be intrinsically linked to the process of disruption, neuroplasticity, and rewiring of neural circuits induced by ECT [13]. However, more research is needed to support this hypothesis.

## Limitations and strengths

The main limitation of our study derives from the sample size of 15 patients with 45 repeated measurements. Yet, as we used NfL as one of the most sensitive biomarkers for neuronal loss [15], any relevant and undetected damage of the central nervous system due to ECT in our longitudinal design can be considered very improbable. It should also be taken into account here that this study could replicate and confirm the main results of our previous study on stability of NfL concentrations in the course of ECT [21], increasing validity and significance of both.

A strength of our study besides using NfL as highly sensitive biomarker is the use of in-depth neuropsychological testing, which allowed for a sensitive monitoring of ECT-induced and clinically relevant cognitive changes, different from less sensitive measures like the MMSE. Future studies might focus on patients with less frequent but even more marked side-effects, e.g., delirium following ECT.

Despite the limitation of a small sample, our study adds to the evidence that ECT-induced cognitive side-effects are not caused by a subtle neuronal damage. This interpretation is in line with previous studies concerning biomarkers for possible damage of the central nervous system in the context of ECT and further underlines the safety of this treatment.

### Supplementary Information

Below is the link to the electronic supplementary material.

Supplementary file1 (DOCX 16 KB)

## Data Availability

The data that support the findings of this study are available from the corresponding author upon reasonable request.
