# Supplementary material for: The myth of brain damage: no change of neurofilament light chain during transient cognitive side-effects of ECT
Source: Eur Arch Psychiatry Clin Neurosci. 2023 Sep 1;274(5):1187–95. doi: 10.1007/s00406-023-01686-8 (PMC11226499; doi:10.1007/s00406-023-01686-8)
Supplement: Supplementary file 1 — Supplementary file1 (DOCX 16 KB) [file 406_2023_1686_MOESM1_ESM.docx]

**Supplementary Table S1** Single cognitive parameters (raw scores and T-values), *N = 15*

| **Cognitive parameters** | **Pre-ECT**  **(*T_1_)*** | | **Post-ECT**  **(*T_2_)*** | | **Follow-up**  **(*T_3_)*** | |
| --- | --- | --- | --- | --- | --- | --- |
|  | Raw scores | T-values | Raw scores | T-values | Raw scores | T-values |
| **Memory** |  |  |  |  |  |  |
| RAVLT sum of learning trials 1-5 | 38.40 ± 12.49 | 36.87 ± 14.32 | 33.27 ± 12.45 | 32.13 ± 14.87 | 42.13 ± 12.69 | 42.80 ± 15.56 |
| RAVLT delayed recall | 6.53 ± 5.22 | 36.73 ± 21.92 | 3.60 ± 4.34 | 26.53 ± 16.22 | 6.00 ± 4.11 | 31.07 ± 17.32 |
| **Executive functions** |  |  |  |  |  |  |
| RWT word fluency (animals) | 18.87 ± 6.41 | 48.87 ± 11.28 | 14.87 ± 7.45 | 41.73 ± 12.57 | 17.00 ± 6.72 | 48.00 ± 13.62 |
| RWT word fluency (s-words) | 11.67 ± 5.29 | 46.27 ± 12.60 | 6.73 ± 5.54 | 34.47 ± 11.26 | 9.80 ± 4.28 | 41.00 ± 11.07 |
| TMT Part B (time in s)^a^ | 172.07 ± 96.16 | 26.71 ± 16.52 | 184.71 ± 93.24 | 26.00 ± 10.09 | 111.79 ± 74.96 | 35.93 ± 15.06 |
| WAIS-IV Digit Span backwards | 7.20 ± 2.21 | 43.47 ± 10.46 | 6.27 ± 2.37 | 39.80 ± 9.92 | 7.33 ± 2.32 | 44.47 ± 9.95 |
| **Attention** |  |  |  |  |  |  |
| TMT Part A (time in s) | 70.60 ± 43.82 | 27.33 ± 11.37 | 75.00 ± 41.53 | 27.67 ± 9.94 | 54.00 ± 21.68 | 31.73 ± 10.38 |
| WAIS-IV Digit Span forwards | 8.53 ± 2.39 | 45.33 ± 11.19 | 7.93 ± 2.12 | 42.60 ± 10.73 | 8.93 ± 2.19 | 47.20 ± 9.13 |

*Notes.* Mean ± standard deviations presented. Abbreviations: RAVLT: German version of the Rey Auditory Verbal Learning Test; RWT: Regensburg Word Fluency Test; TMT: Trail Making Test; WAIS-IV: Wechsler Adult Intelligence Scale – fourth edition. ^a^*n* = 14 due to missing data.
